# Validity between signs and symptoms of sleep bruxism against a validated portable electromyographic device 

**DOI:** 10.4317/jced.61720

**Published:** 2024-11-01

**Authors:** Márcio Lima Grossi, Lourenço Oliveira Castillo, Marcos Pascoal Pattussi, Georgia Meneghini Pinto, Ruy Teichert Filho

**Affiliations:** 1 DDS, MS, PhD, Professor, Post-Graduate Program in Dentistry (Prosthodontics), Pontifical Catholic University of Rio Grande do Sul (PUCRS), Professor, Porto Alegre, Brazil; 2 DDS, MS, PhD. Post-Graduate Program in Dentistry (Prosthodontics), Pontifical Catholic University of Rio Grande do Sul (PUCRS), Professor, Porto Alegre, Brazil; 3 Associate Professor, Post-Graduate Program in Community Health, Universidade do Vale do Rio dos Sinos – UNISINOS, São Leopoldo, RS; 4 DDS, Specialist Orthodontics, School of Health and Life Sciences Course in Dentistry. Pontifical Catholic University of Rio Grande do Sul (PUCRS); 5 DDS, MS, PhD. Post-Graduate Program in Dentistry (Prosthodontics), Pontifical Catholic University of Rio Grande do Sul (PUCRS), Porto Alegre, Brazil

## Abstract

**Background:**

Sleep bruxism is a major research area in dentistry today and needs valid clinical means of diagnosis against valid instrumental methods. Purpose: To assess the validity of the most commonly reported sleep bruxism (SB) signs and symptoms in the literature against a polysomnography (PSG) validated portable electromyographic (EMG) device (BiteStrip®).

**Material and Methods:**

Fifty young adults (40 women & 10 men, 18-30 years old) volunteered for the sequential and simultaneous administration of the SB signs and symptoms questionnaire versus the BiteStrip®. The SB signs and symptoms questionnaire was comprised of 19 items divided in 5 areas: a) Area 1: self-awareness of tooth grinding, clenching, and/or tooth sounds/noises, b) Area 2: headaches and/or facial pain, c) Area 3: muscle fatigue and/or hypertrophy, d) Area 4: clicking, crepitation and/or locking in the TMJ, and e) Area 5: tooth sensitivity, tooth wear/breaking, and/or cheek/tongue indentations. A cross-tabulation between the dichotomic test results (positive = 1, negative = 0) between the all five SB areas separately using quartiles (positive test result=75th percentile or higher, negative test result=50th percentile or lower) versus a positive test result of the BiteStrip® (score=1 or higher) was performed.

**Results:**

Area 1 presented the highest sensitivity for SB screening (80.0%), but with low specificity (51.4%), diagnosing most SB cases, but with a high number of false positives. All other four areas had low sensitivity (range=37.9% to 58.6%) and screening capacity and are only useful if Area 1 is positive.

**Conclusions:**

Commonly reported SB signs and symptoms are not valid diagnostic measurements and can only be used as a screening method for either ‘possible’ or ‘probable’ SB diagnosis.

** Key words:**Sleep bruxism, electromyography, validation study, polysomnographies.

## Introduction

A recent conclusion by a panel of experts suggested that bruxism diagnosis can be divided into three different categories: ‘possible’ sleep/awake bruxism based on a positive self-report only; ‘probable’ sleep/awake bruxism based on a positive clinical inspection, with or without a positive self-report; and ‘definite’ sleep/awake bruxism based on a positive instrumental assessment, with or without a positive self-report, and/or a positive clinical inspection (1-3). The general clinical recommendation for SB diagnosis is that self-reports and clinical examination in combination can be performed for daily practice; however, in order to confirm the clinical SB diagnosis, instrumental diagnoses (validated porTable EMG instruments against PSG) should be used. At the present time, two portable EMG/ECG appliances have been successfully tested against PSG (BiteStrip® and Bruxoff®) for subjects with positive history of SB (screening) (4-6). However, they are indicated only for primary SB; when sleep, neurological, or other systemic disorders affecting sleep are not present (4-6).

To these authors’ knowledge, a validity study comparing most of the reported signs and symptoms of SB, including both patient history and clinical examination, against a PSG validated porTable EMG instrument is still needed in the literature (1-3).

## Material and Methods

-Study design and ethical approval

The objective of this study was to assess the validity of the most commonly reported SB signs and symptoms from the questionnaires of the specialized literature against a porTable EMG device validated against PSG (BiteStrip®).

-Population and study protocol

The SB signs and symptoms questionnaire was applied in 50 young adults (non-patient population) of both sexes recruited from internet and printed adds at the Pontifical Catholic University of Rio Grande do Sul. In order to be enrolled, subjects needed not to self-report chronic pain, not to be under treatment for systemic or psychiatric diseases, and not to be under the use of medications affecting the central nervous system. All volunteers underwent the SB signs and symptoms questionnaire self-assessment and clinical examination on the same night they used the EMG porTable device (BiteStrip®) (7,8). The EMG assessment was performed in the patient’s home environment (2). A single experienced examiner in charge of the clinical examination (questions 6, 10 and 11, only) was different and blinded from the one providing both the BiteStrip® instructions and the SB self-completing questionnaire (7).

-Development, completion and scoring of the SB signs and symptoms questionnaire

The SB questionnaire questions were selected from the literature and grouped in five areas of screening and diagnostic interest: a) Area 1: presence of bruxism activity self-awareness, such as: clenching, grinding, tooth sounds/noises during the night for the detection SB (9-14); b) Area 2: presence of orofacial pain symptoms, such as the presence of headaches and/or facial pain upon awakening (5,8,15-20); c) Area 3: presence of signs and symptoms of masticatory muscle dysfunction, such as masticatory muscle fatigue upon awakening and/or hypertrophy (3,5,8,18-22); d) Area 4: presence of signs and symptoms of temporomandibular joint (TMJ) dysfunction, such as clicking, crepitation and/or locking in the TMJ upon awakening (5,21,22); and e) Area 5: self-awareness of the presence of tooth sensitivity upon awakening, tooth and/or restoration wear/breaking, and/or cheek/tongue indentations (3,8,16,17,19,21).

Regarding the quantitative scoring, the SB signs and symptoms questionnaire is comprised by 11 questions divided into 5 areas described above, which described the presence and/or absence of SB signs and symptoms. With the exception of questions 6, 10 and 11, all other questions have sub-items to assess frequency (three times per week) of signs and symptoms of SB, resulting in a total of 19 questions, where each positive answer = 1 point. A total score of 19 points in five areas is divided by: a) Area 1 (questions 1a, 1b, 2a, 2b) = 4 possible points, b) Area 2 (questions 3a, 3b, 4a, 4b) = 4 possible points, c) Area 3 (questions 5a, 5b, 6) = 3 possible points, d) Area 4 (questions 7a, 7b, 8a, 8b) = 4 possible points, and e) Area 5 (questions 9a, 9b, 10, 11) = 4 possible points ( Table 1, Table 2, Table 3). Only questions 6 (face muscle hypertrophy), 10 (wearing and/or braking of teeth and/or restorations), and 11 (cheek and/or tongue tooth marks), in  Table 2 and  Table 3, were confirmed by extra and intra-oral clinical examinations (present or absent) (1).

-Bruxism diagnosis using the BiteStrip®

The BiteStrip® is a porTable surface EMG device, and it has a computer chip that registers the number of contractions or bursts (30% or more of maximum voluntary clenching) of the masseter muscle (SB) during five hours of sleep time. This EMG validated against PSG device was placed on the left masseter only and employed according to the manufacturer’s instructions (4,5). According to the manufacturer, contractions that exceed 30% of the maximum voluntary clenching muscle activity was considered a SB episode.

-Sample size and statistical analysis

The statistical analysis was performed using the SPSS 22.0 (IBM Co., Armonk, NY, EUA). The SB signs and symptoms questionnaire diagnostic validity against the BiteStrip® was assessed by the following diagnostic measures: sensitivity, specificity, positive and negative predictive values (PPV and NPV), area under a receiver operating curve (ROC), and positive and negative likelihood ratio (PLR and NLR) (23). The sample size was calculated for a SB screening device (high sensitivity or high true positives), yielding a total = 49 subjects; 20 subjects were added to compensate for drop-outs, totaling = 69 subjects (24).

## Results

A total of 78 subjects volunteered for the study, but 28 non-eligible subjects were excluded. Fifty young adults (20.78±2.57 years old, range=18-30), 40 women and 10 men, were eligible and sequentially enrolled to participate in the study between 2017 and 2019. All subjects that agreed to participate had no missing data in any of the questions of the SB signs and symptoms questionnaire and no complaints or missing data in the use of the BiteStrip®. 

 Table 1 analyzed Area 1, which detected the self-awareness of clenching, grinding, and/or tooth sounds during the night (questions 1a, 1b, 2a, and 2b), and it showed a prevalence of SB ranging from 8% to 20%, if we consider only questions 1b and 2b. These two questions assessed the frequency of SB self-awareness (three or more times a week) after a positive SB response (presence and/or absence) in questions 1a and 2a.

 Table 2 analyzed both Areas 2 and 3. In Area 2, questions 3a and 3b analyzed the presence/absence and the frequency of headaches upon awakening lasting more than 30 minutes for three or more times a week, respectively; yielding a prevalence of 4% (3b only). Questions 4a and 4b analyzed the presence/absence and the frequency of pain in the face upon awakening lasting more than 30 minutes for three or more times a week, respectively; yielding a prevalence of 12% (4b only). In Area 3, questions 5a and 5b analyzed the presence/absence and frequency of tiredness (fatigue) of jaw muscles upon awakening lasting three or more times a week respectively, yielding a prevalence of 12% (5b only). Question 6 analyzed, in a single question, the presence/absence of the self-awareness that the face muscles are increased in size, confirmed by clinical examination, yielding a prevalence of 16%.

 Table 3 analyzed both Areas 4 and 5. In Area 4, questions 7a, 7b, 8a, and 8b analyzed the presence/absence and the frequency (three times a week) of signs and symptoms of TMJ dysfunction (clicking, crepitus, and locking) upon awakening. Clicking and/or locking of the TMJ for more than three times a week yielded a prevalence of 12% (7b only); while crepitus and/or locking for more than three times a week yielded a prevalence of 4% (8b only). In Area 5, questions 9a and 9b analyzed the presence/absence and the frequency (three times a week) of tooth sensitivity upon awakening, respectively, which yielded a prevalence of 4% (9b only). Question 10 analyzed, in a single question, the presence/absence of tooth and/or restoration wearing and/or breaking, yielding a prevalence of 12%. Question 11 analyzed, in a single question, the presence of tooth marks in the tongue and/or cheeks, yielding a prevalence of 30.6%. Both questions 10 and 11 were confirmed by clinical examination.

In  Table 4, the validity of the SB signs and symptoms questionnaire was assessed by a cross tabulation with the BiteStrip® results. The qualitative analyses were performed by a cross-tabulation between the dichotomic test results (positive = 1, negative = 0) between the SB signs and symptoms questionnaire (assessment of all five areas individually) versus the BiteStrip®, which was dichotomized according to its display results (score 0 = no bruxism; scores 1, 2 or 3 = positive bruxism).

The five individual areas of the SB signs and symptoms questionnaire were separately dichotomized according to their respective quartiles (25th, 50th, and 75th percentiles). A positive test result for each individual area of the questionnaire was considered in the 75th percentile or higher, while a negative test result was considered the 50th percentile or lower, for the total number of positive answers in that particular area (4). According to our results, this granted the following SB signs and symptoms questionnaire positive test results for each area separately: a) Area 1: 1 positive out of 4 possible answers, b) Area 2: 2 positive out of 4 possible answers, c) Area 3: 1 positive out of 3 possible answers, d) Area 4: 1 positive out of 4 possible answers, and e) Area 5: 1 positive out of 4 possible answers.

The results of all measures of validity assessed in this study (sensitivity, specificity, ROC, PPV, NPV, PLR, and NLR) clearly showed that none of the five SB signs and symptoms questionnaire areas, when analyzed individually ( Table 4), reached acceptable results (70.0%) for sensitivity and specificity, including Area 1 (23). Area 1 presented the best screening results for SB among all five areas assessed with high sensitivity (80.0%) and with low specificity (51.4%), diagnosing most SB cases, but with a high number of false positives. All other four areas (Areas 2 to 5) had low sensitivity (range=37.9% to 58.6%) and had low capacity to screen for SB positive cases and can be used only in combination with a positive SB test result in Area 1.

## Discussion

In Area 1 ( Table 1), the prevalence distribution of SB self-awareness three or more times a week in this study ranged from 8% to 20%. This is in agreement with a systematic review which found that the SB prevalence varied according to the diagnostic method used: a) 22% to 29% in self-reported questionnaires (i.e., ‘possible’ bruxism); b) 10% to 23% in clinical assessment (i.e., ‘probable’ bruxism); and c) 0.14% to 8% in porTable EMG diagnostic devices (i.e., ‘definite’ bruxism) (2).

In Area 2 ( Table 2), the presence and frequency of headaches upon awakening lasting more than 30 minutes for three or more times a week yielded a prevalence of 4%. This is important, because adult patients with SB complain of headache more frequently in the morning upon awakening (9), and provides an indication of the severity of bruxism and possible comorbidity with sleep apnea (14). Pain the face upon awakening lasting more than 30 minutes for three or more times a week gave a prevalence of 12%, but the relationship between TMD pain and SB is still contradictory (25).

In Area 3 ( Table 2), 12% of subjects reported self-awareness of tiredness (fatigue) upon awakening lasting three or more times a week; while 16% reported and confirmed by clinical examination that the face muscles were increased in size. Masticatory muscle dysfunction different from pain has been recently substantiated by a recent systematic review with 51 studies, which despite not showing a direct correlation with SB, provided data to support that non-pain musculoskeletal symptoms might precede TMD pain (26).

In Area 4 ( Table 3), 12% of subjects reported jaw joint clicking sounds and 4% reported cracking or rusting sounds, both with limitation or locking during opening upon awakening three or more times a week. Nevertheless, two recent studies found no significant association between SB with TMJ clicking (27,28).

In Area 5 ( Table 3), 4% of subjects reported tooth sensitivity to cold, heat, or sweat upon awakening three or more times a week. In addition, 12% reported, confirmed by clinical examination, that the front and/or back teeth/restorations are wearing out and/or braking too fast. The recent literature showed that a negative correlation was found between tooth wear and both SB rhythmic masticatory muscle activities (RMMAs) and sleep-time masseter muscle activity (sMMA) (29). In a review study, other factors (age, occlusal conditions, enamel characteristics, diet, carbonated drinks, medications, gastroesophageal reflux disease, and alimentary disorders) might be related to tooth attrition and erosion (20). Similarly, tooth marks in the tongue and/or cheeks had a prevalence of 30.6% in all subjects confirmed by clinical examination. Literature suggests that tooth indentations might be related more to anatomical and physiological factors of the tongue itself (width at rest) than to SB (30). In a review, both masseter hypertrophy and tooth indentations might be also associated with daytime-wake oral parafunctions (tooth clenching, tongue pushing, and excessive swallowing) (31).

The results of all measures of validity assessed in this study ( Table 4) demonstrated that none of the five SB signs and symptoms questionnaire areas, when analyzed individually, reached acceptable results, including Area 1 (24). Area 1 presented the best screening results for SB among all five areas assessed with high sensitivity and low specificity, diagnosing 80% of the SB cases, but with a high number of false positives. The other four areas (2 to 5) had low sensitivity and consequently low capacity to screen for SB positive cases, and they should be used only in combination with a positive SB positive result in Area 1.

## Conclusions

The SB signs and symptoms questionnaire Area 1 (self-report of clenching, grinding, tooth sounds/noises) qualitative score demonstrated to be useful only as a screening questionnaire for SB as either ‘possible’ or ‘probable’ diagnosis when compared to a porTable EMG device (BiteStrip®), which was validated against PSG. Other SB signs and symptoms assessed in the SB signs and symptoms questionnaire (Areas 2 to 5) are only useful in the presence of a positive result for Area 1.

## Figures and Tables

**Table 1:** Prevalence of a dichotomous test result (0 = negative; 1 = positive) for sleep bruxism using the SB signs and symptoms questionnaire in Area 1 (N = 50).

	Negative Result = 0 Positive Result = 1	N	%
Area 1^†^: self-awareness of clenching, grindings, tooth sounds/noises during the night			
(1a) Have you ever been told that you grind and/or clench your teeth producing sounds/noises at night while sleeping?	No = 0 Yes = 1	37 13	74.0 26.0
(1b) If yes, how many times a week?	1 or 2 times = 0 3 or more times = 1	46 4	92.0 8.0
(2a) Have you ever noticed that you grind and/or clench your teeth at night while sleeping?	No = 0 Yes = 1	28 22	56.0 44.0
(2b) If yes, how many times a week?	1 or 2 times = 0 3 or more times = 1	40 10	80.0 20.0

† Positive result for Area 1 (1a+1b+2a+2b) = 1 positive answer out of 4 possible answers.

**Table 2:** Prevalence of a dichotomous test result (0 = negative; 1 = positive) for sleep bruxism using the SB signs and symptoms questionnaire in Areas 2 and 3 (N = 50).

	Negative Result = 0 Positive Result = 1	N	%
Area 2^†^: presence of headaches and/or orofacial pain			
(3a) Have you ever felt headaches upon awakening which lasted between 30 minutes and 4 hours?	No = 0 Yes = 1	32 18	64.0 36.0
(3b) If yes, how many times a week?	1 or 2 times = 0 3 or more times = 1	48 2	96.0 4.0
(4a) Have you ever felt pain in your face upon awakening?	No = 0 Yes = 1	29 21	58.0 42.0
(4b) If yes, how many times a week?	1 or 2 times = 0 3 or more times = 1	44 6	88.0 12.0
Area 3^‡^: presence of signs and symptoms of masticatory muscle dysfunction			
(5a) Have you ever felt tiredness in your jaw upon awakening?	No = 0 Yes = 1	28 22	56.0 44.0
(5b) If yes, how many times a week?	1 or 2 times = 0 3 or more times = 1	44 6	88.0 12.0
(6) Have you noticed that your face muscles are increased in size?	No = 0 Yes = 1	42 8	84.0 16.0

† Positive result for Area 2 (3a+3b+4a+4b) = 2 positive answers out of 4 possible answers. ‡Positive result for Area 3 (5a+5b+6) = 1 positive answer out of 3 possible answers.

**Table 3:** Prevalence of a dichotomous test result (0 = negative; 1 = positive) for sleep bruxism using the SB signs and symptoms questionnaire in Areas 4 and 5 (N = 50).

	Negative Result = 0 Positive Result = 1	N	%
Area 4^†^: presence of signs and symptoms of temporomandibular joint (TMJ) dysfunction			
(7a) Have you ever felt jaw joint clicking sounds with limitation or locking during opening upon awakening?	No = 0 Yes = 1	36 14	72.0 28.0
(7b) If yes, how many times a week?	1 or 2 times = 0 3 or more times = 1	44 6	88.0 12.0
(8a) Have you ever felt jaw joint cracking or rusting sounds with limitation or locking during opening upon awakening?	No = 0 Yes = 1	44 6	88.0 12.0
(8b) If yes, how many times a week?	1 or 2 times = 0 3 or more times = 1	48 2	96.0 4.0
Area 5^‡^: presence of tooth sensitivity and tooth wear			
(9a) Have you ever felt tooth sensitivity to cold, heat, or sweat upon awakening?	No = 0 Yes = 1	38 12	76.0 24.0
(9b) If yes, how many times a week?	1 or 2 times = 0 3 or more times = 1	48 2	96.0 4.0
(10) Have you ever noticed that your front and/or back teeth/restorations are wearing out and/or braking too fast?	No = 0 Yes = 1	44 6	88.0 12.0
(11) Have you ever noticed that you have tooth marks in your tongue and/or cheeks?	No = 0 Yes = 1	34 15	69.4 30.6

† Positive result for Area 4 (7a + 7b + 8a + 8b) = 1 positive answer out of 4 possible answers. ‡Positive result for Area 5 (9a + 9b + 10 + 11) = 1 positive answer out of 4 possible answers.

**Table 4:** Comparison of a dichotomous test result (0 = negative; 1 = positive) for sleep bruxism comparing the SB signs and symptoms questionnaire † versus the BiteStrip ‡, (N = 50) in Areas 1 to 5.

Diagnostic Test Result (N = 50)	Area 1 (a) % (95% CI)	Area 2 (b) % (95% CI)	Area 3 (c) % (95% CI)	Area 4 (d) % (95% CI)	Area 5 (e) % (95% CI)
Sensitivity	80.0 (58.3 - 100.0)	41.4 (23.5 - 61.1)	58.6 (38.9 - 76.5)	37.9 (20.7 - 57.7)	58.6 (38.9 – 76.5)
Specificity	51.4 (35.3 - 67.7)	85.7 (63.7 – 97.0)	66.7 (43.0 – 85.4)	81.0 (58.1 – 94.6)	61.9 (38.4 – 81.9)
Receiver Operating Curve (ROC)	64.0 (48.0 - 79.0)	63.5 (51.6 – 75.5)	62.6 (48.9 – 76.4)	59.4 (47.0 – 71.9)	60.3 (46.2 – 74.3)
Positive Predictive Value	41.4 (23.3 - 60.7)	80.0 (51.9 – 95.7)	70.8 (48.9 – 87.4)	73.3 (44.9 – 92.2)	68.0 (46.5 – 85.1)
Negative Predictive Value	85.7 (68.2 - 100.0)	51.4 (34.0 - 68.6)	53.8 (33.4 – 73.4)	48.6 (31.4 - 66.0)	52.0 (31.3 – 72.2)
Positive Likelihood Ratio	1.6 (1.0 - 2.6)	2.9 (0.9 - 9.0)	1.8 (0.9 - 3.5)	2.0 (0.7 – 5.4)	68.0 (46.5 – 85.1)
Negative Likelihood Ratio	0.4 (0.0 - 0.9)	0.7 (0.5 – 1.0)	0.6 (0.4 - 1.0)	0.8 (0.5 – 1.1)	52.0 (31.3 – 72.2)

† The sleep bruxism signs and symptoms questionnaire was dichotomized according to the percentile distribution (quartiles) of the data in each of the five areas (i.e., 50th or lower = negative, and 75th or higher = positive). (a) Area 1: clenching, grinding, tooth sounds/noises during the night, (b) Area 2: headaches and/or facial pain, (c) Area 3: masticatory muscle fatigue or hypertrophy, (d) Area 4: clicking, crepitation and/or locking in the temporomandibular joint, and (e) Area 5: tooth grinding, tooth sensitivity or cheek/lip indentations. ‡ Bitestrip was dichotomized according to its display results (score 0 = no bruxism; scores 1, 2 or 3 = positive Bruxism.

## Data Availability

The datasets used and/or analyzed during the current study are available from the corresponding author.
